# The association between breastfeeding and childhood obesity/underweight: a population-based birth cohort study with repeated measured data

**DOI:** 10.1186/s13006-022-00522-4

**Published:** 2022-12-01

**Authors:** Weiqin Li, Jiarong Yuan, Leishen Wang, Yijuan Qiao, Enqing Liu, Shuo Wang, Junhong Leng

**Affiliations:** Tianjin Women’s and Children’s Health Center, Tianjin, 300070 China

**Keywords:** Breastfeeding, Children, Epidemiology, Obesity, Underweight

## Abstract

**Background:**

The impact of breastfeeding on childhood obesity has long been under debate, with most research showing significant association, and others showing weak or no association between breastfeeding and childhood obesity. What’s more, almost all of the previous studies focused on the association between breastfeeding and childhood obesity, and no studies have assessed the association between breastfeeding and childhood underweight.

This study aimed to examine the association between breastfeeding and childhood obesity as well as childhood underweight from 1 to 6 years old.

**Methods:**

A retrospective population-based cohort study of 59,564 children born between May 2009 and April 2013 in China was conducted using the healthcare records data from the Tianjin Maternal and Child Healthcare System. Information on infant breastfeeding (exclusive breastfeeding, mixed feeding, and exclusive formula feeding) within 6 months old and childhood growth (6 times of repeated measured weight and height from 1 to 6 years old) was collected. Multinomial logistic regression was used to test the potential associations between infant feeding modalities and childhood growth (underweight, normal weight and obesity).

**Results:**

Compared with exclusive formula feeding, breastfeeding was inversely associatied with childhood obesity from 2 to 6 years old, and there was a trend from mixed feeding to exclusive breastfeeding (*P*_trend_ < 0.05). The largest association with obesity was displayed at 3 years old, with the multivariable adjusted odds ratios (ORs) for exclusive formula feeding, mixed feeding and exclusive breastfeeding of 1.00, 0.62 (95% CI 0.49, 0.80) and 0.57 (95% CI 0.44, 0.74) (*P*_trend_ = 0.001), respectively. Compared with exclusive breastfeeding, exclusive formula feeding may increase the risk of childhood underweight at 3 and 5 years old.

**Conclusions:**

Breastfeeding was inversely associated with the risk of childhood obesity from 2 to 6 years old, and there was a trend from mixed feeding to exclusive breastfeeding. Infant exclusive formula feeding might be a risk factor for childhood underweight at preschool time.

## Background

Childhood obesity has dramatically increased, and it is now considered a global problem [1]. In 2013, the prevalence of overweight/obesity in children and adolescents was 24% of boys and 23% of girls in developed countries, and 13% of both boys and girls in developing countries [1]. In China, the prevalence of overweight and obesity in children increased from 5% in 1995 to 21% in 2014 [2]. Childhood obesity is a strong predictor of adult obesity, and is linked to metabolic outcomes in childhood, adolescence, and adulthood [3–5].

Besides overweight and obesity, undernutrition (wasting, stunting, and underweight) is another form of malnutrition. The World Health Organization (WHO) reported that in 2016, 47 million children under 5 years of age are wasted, 14.3 million are severely wasted and 144 million are stunted [6]. Combating malnutrition in all its forms is one of the greatest global health challenges. Optimizing nutrition early in life, including the 1000 days from conception to a child’s second birthday, ensures the best possible start in life, with long-term benefits [6].

The impact of breastfeeding is currently attracting considerable interest. Children and adults who were breastfed have been found to have lower rate of diabetes, and perform better on intelligence tests than persons who were formula-fed [7]. Current data on the impact of breastfeeding on childhood overweight provide equivocal, with most research showing significant association [8, 9], and others showing weak or no association between breastfeeding and childhood obesity [10]. To our knowledge, no studies have focused on the effect of breastfeeding on underweight in childhood.

Using the data of a population-based cohort study with a large sample size, the aim of the present study was to examine the association between breastfeeding and childhood growth from 1 to 6 years of age in China.

## Methods

A population based cohort study was conducted based on the healthcare records data from the Tianjin Maternal and Child Healthcare System in Tianjin, China.

Tianjin is a metropolitan city in Northern China, ranking fourth in population size (15 millions) among Chinese cities. It consists of 16 county-level administrative areas, including 6 central urban districts, 1 new urban district, 6 suburban districts, and 5 rural districts. Tianjin Maternal and Child Healthcare System, which is managed by Tianjin Women and Children’s Health Center (WCHC), is a 3-tier care system, consisting of 1) About 300 community-based health centers and 1700 kindergartens; 2) 6 district-level WCHC and other secondary obstetric hospitals; and 3) A city-level Tianjin WCHC and other tertiary obstetric hospitals. In Tianjin, all women’s antenatal care and children’s health care were delivered in a relatively structured manner [11, 12], and health care records for both pregnant women and children have been collected and available in electronic form since 2009. All pregnant women register at a community-based health center with regular antenatal clinic visits until 28^th^ gestational week, and then they are referred to one of the secondary or tertiary obstetric hospitals at their choice where they will be managed till delivery. Information of antenatal care for women include general information (birth date, ethnicity, education, prepregnancy body weight, abortion history, last menstrual period, smoking and drinking habits, etc.) which are self-reported at the first antenatal care visit, clinical measurements (height, weight, and blood pressure for each antenatal care visit, gynecological examinations, ultrasonography, gestational diabetes screening result at 24–28 gestational week, and other lab tests), pregnancy outcomes (date of delivery, delivery modes, labor complications, etc.), and postnatal period examinations (< 42 days after delivery). All children are given health examinations by the community-based health centers at birth, during postnatal period (< 42 days after birth), and at infancy and early childhood; and then by the district-level or city-level WCHC after they enter kindergartens. Information of health examinations for children begin with children’s birth, including information from newborns (date of birth, sex, gestational week of birth, birth weight, birth recumbent length, and Apgar score), postnatal period (names of the child and his/her parents, family history of diseases, feeding modalities, weight, and recumbent length), infancy (date of examination, weight, recumbent length before 24 months and height from 24 months, head circumference, number of teeth, and blood hemoglobin), and preschool (date of examination, weight, height, number of teeth, blood hemoglobin, and blood pressure), as well as the information of feeding modalities (exclusive breastfeeding, mixed feeding, and exclusive formula feeding) during the first 6 months.

## Study sample

Between May 1, 2009 and April 30, 2013, a total of 197,938 children were registered in Tianjin Maternal and Child Healthcare System and attended their primary health care from birth to preschool. Of them, there were 69,749 children with all the information of weight and length/height at ages of 1, 2, 3, 4, 5, and 6 years old. After excluding 10,185 children who were multiple births (*n* = 777) or with incomplete information of feeding modalities during the first 6 months (*n* = 9,408), the present study included 59,564 children (30.1%) with all the key information needed.

The Ethics Committee for Clinical Research of Tianjin Women and Children’s Health Center approved the study and analysis plan. Tianjin Women and Children’s Health Center agreed to waive the need for written informed consent from all participants involved in our study because we used the electronic dataset from health care records.

## Measurements

The dataset for the present analyses was from electronic health care records of the annual regular health examination. No patients were involved in this study. The dataset included maternal characteristics (maternal age at delivery, prepregnancy body weight, education, smoking status, and gestational diabetes at term preganancy), and child characteristics [sex, and information at birth (birth weight, birth length and gestational age), feeding modalities (exclusive breastfeeding, mixed feeding, and exclusive formula feeding at 6 months old), and weight and height/length at 1, 2, 3, 4, 5 and 6 years old].

Children’s anthropometric data, including body weight, height, or recumbent length (before 24 months), were collected at birth, the 1^st^ year (0.76 ~ 1 years), 2^nd^ year (1.51 ~ 2 years), 3^rd^ year (2.51 ~ 3 years), 4^th^ year (3.51 ~ 4 years), 5^th^ year (4.51 ~ 5 years), and 6^th^ year (5.51 ~ 6 years). Using the standardized protocol, all children’s weight and length/height were measured in light indoor clothing and without shoes by specially-trained pediatricians in community health centers. Weight was measured to the nearest 0.01 kg by using a digital scale (TCS-60, Tianjin Weighing Apparatus Co., China). Length was measured to the nearest 0.1 cm by using a recumbent length stadiometer (YSC-2, Beijing Guowangxingda, China). Standing height was measured to the nearest 0.1 cm using a stadiometer (SZG-180, Shanghai Zhengdahengqi, China). The electronic healthcare records were verified with repeated measurements previously [13]. The correlations between electronic healthcare records and repeated measurement data for children are 0.999 (for body weight) as well as 0.999 (for height/recumbent length), respectively.

Body mass index (BMI) was calculated by dividing weight in kilograms by the square of height in meters. Z scores (standard deviation [SD] scores) were calculated independent of sex and age—that is, (measurement minus population mean) /population SD. BMI z scores, representing equivalent BMI-for-age percentile, were calculated based on the WHO child growth standards (2 ~ 5 years old) and WHO child growth reference (5 ~ 19 years old). According to BMI z scores, children’s body size was divided into 3 categories: < -2SD, -2SD to 2SD, and ≥ 2SD for underweight, normal weight and obesity, respectively.

When the infant attended the 6-month-old routine physical examination, his/her parents or guardians were asked how the infant was fed (exclusive breastfeeding, mixed feeding, or exclusive formula feeding). Exclusively breastfeeding means no other foods or liquids are provided including water since 24 h. The response was recorded in the Tianjin Maternal and Child Healthcare System.

## Statistical analyses

One-way analysis of variance and chi-square test (continuous and categorical variables, respectively) were used to compare the general characteristics of both mothers and children, according to infant feeding modalities (exclusive formula feeding, mixed feeding, and exclusive breastfeeding). As the outcome variable, childhood growth (underweight, normal weight and obesity), is of 3 categories, multinomial logistic regression was used to test the potential risk factors including infant feeding modalities. In the multinomial logistic regressions, 2 logistic functions were conducted, one with underweight compared to normal weight, and the other with obesity compared to normal weight. A structured adjustment scheme was used to consider confounding effects of other covariables. Three models were used in the logistic analyses: Model 1 univariate analyses; Model 2 adjusted for sex, gestational age, birth weight and maternal characteristics (age, education, smoking status, and gestational diabetes in term pregnancy); Model 3 adjusted for covariates in Model 2 and also for maternal prepregnancy BMI.

This study is a longitudinal design in which the same dependent variable (obesity, normal weight and underweight) is repeatedly measured over time (1, 2, 3, 4, 5, and 6 years old) on the same children. And the repeated measured data is a categorical dependent variable. Marginal Models (generalized estimating equation, GEE) were conducted to investigate changes in the dependent variable over time and to compare these changes across groups of infant feeding modalities [14]. The multiplicative interaction between time (age) and infant feeding modalities was also tested. In this analysis, infant feeding modality was transferred to a binary variable, breast feeding (both of exclusive breastfeeding and mixed feeding) and exclusive formula feeding.

The criterion of statistical significance was < 0.05 (for 2-sided tests). All statistical analyses used SAS for Windows, version 9.3 (SAS Institute, Cary, NC).

## Results

Of the 59,564 children, the rates of exclusive formula feeding, mixed feeding, and exclusive breastfeeding at six months were 10.0%, 59.4% and 30.6%, respectively. The general maternal and child characteristics were all significantly different according to infant feeding modalities as listed in Table 1.

**Table 1:** Characteristics of mother–child pairs according to infant feeding modalities

	Exclusive formula feeding	Mixed feeding	Exclusive breastfeeding	*P* value
Number of subjects (%)	5934 (10.0)	35,374 (59.4)	18,256 (30.6)	
***Maternal characteristics***				
Maternal age at term delivery, years	28.1 ± 4.1	27.6 ± 4.3	27.9 ± 4.2	< 0.001
Prepregnancy BMI category, %				0.002
< 18.5 kg/m^2^	15.5	14.0	13.2	
18.5 ~ 23.9 kg/m^2^	60.6	63.3	64.4	
24.0 ~ 27.9 kg/m^2^	17.0	17.2	16.9	
≥ 28.0 kg/m^2^	6.9	5.5	5.4	
Education, %				< 0.001
≤ 9 years	18.1	30.3	25.6	
> 9 ~ ≤ 12 years	20.4	18.6	17.5	
> 12 ~ ≤ 16 years	58.5	47.6	51.7	
> 16 years	3.0	3.5	5.2	
Smokers, %	1.4	1.2	0.9	0.001
Gestational diabetes at term pregnancy, %	7.0	4.9	5.5	< 0.001
***Child characteristics***				
Sex, %				< 0.001
Boys	54.6	51.6	51.7	
Girls	45.4	48.4	48.3	
Birth weight, g	3347 ± 493	3390 ± 447	3393 ± 440	< 0.001
Birth weight category, %				< 0.001
< 2.5 kg	3.7	1.9	1.6	
2.5 ~ < 4.0 kg	88.9	91.1	91.3	
≥ 4.0 kg	7.4	7.1	7.1	
Gestational age at delivery, weeks	39.4 ± 1.6	39.6 ± 1.5	39.6 ± 1.4	< 0.001
Z-scores for body mass index for age				
1 Years old	0.69 ± 1.0	0.69 ± 1.0	0.63 ± 1.0	< 0.001
2 Years old	0.64 ± 1.0	0.62 ± 1.0	0.56 ± 1.0	< 0.001
3 Years old	0.32 ± 1.1	0.31 ± 1.0	0.26 ± 1.0	< 0.001
4 Years old	0.22 ± 1.1	0.22 ± 1.1	0.19 ± 1.1	0.012
5 Years old	0.21 ± 1.2	0.23 ± 1.1	0.20 ± 1.1	0.035
6 Years old	0.28 ± 1.4	0.29 ± 1.3	0.26 ± 1.3	0.038

Data were means ± SDs or percentage

**Fig. 1:**
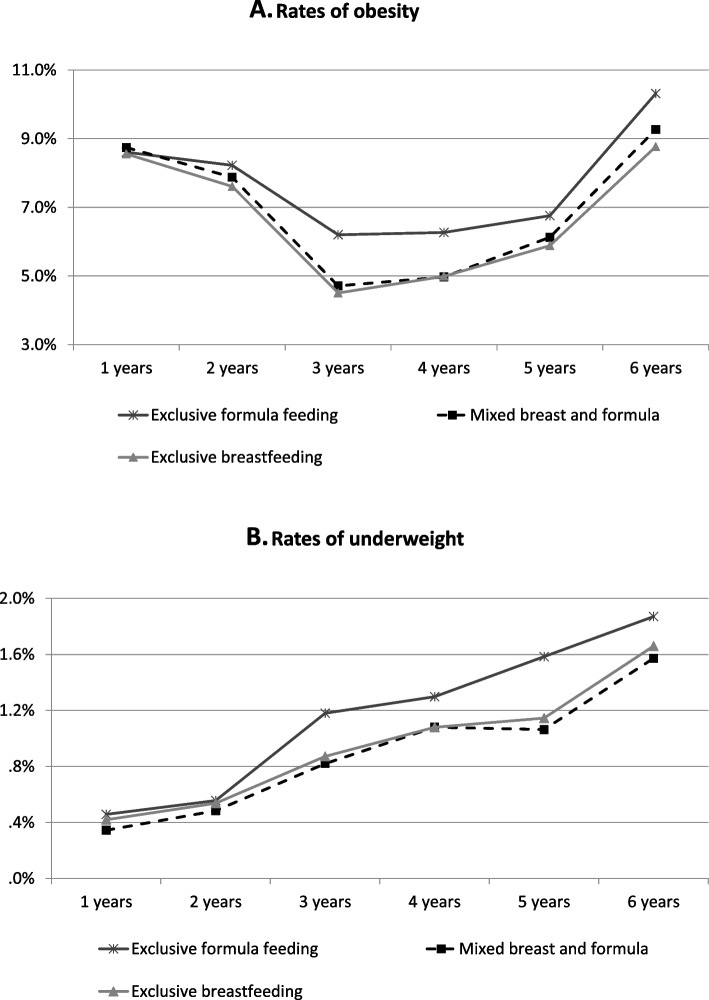
The rates of obesity (**A**) and underweight (**B**) stratified by infant feeding modalities with 6 times of repeated measured data from 1 to 6 years old. Underweight, normal weight and obesity are defined as body mass index < -2SD, -2SD to 2SD, and ≥ 2SD, respectively

Figure 1 presents the rates of obesity and underweight from 1 to 6 years old according to infant feeding modalities. For all 3 kinds of infant feeding modalities, the rates of obesity decreased first and then increased after 3 years old, while the rates of underweight increased as the children grew up from 1 to 6 years. Compared with those who were exclusively formula fed, children fed by mixed breast and formula or exclusive breast had lower rates of obesity as well as underweight from 3 to 6 years old.

Compared with exclusive formula feeding, breastfeeding was inversely associatied with childhood obesity from 2 to 6 years old, and there was a trend from mixed feeding to exclusive breastfeeding (P_*trend*_ < 0.05). The largest association with obesity was displayed at 3 years old, with the multivariable adjusted odds ratios (ORs) for exclusive formula feeding, mixed feeding and exclusive breastfeeding of 1.00, 0.62 (95% CI 0.49, 0.80) and 0.57 (95% CI 0.44, 0.74) (P_*trend*_ = 0.001), respectively (Model 3, Table 2).

**Table 2:** Odds ratios (ORs) of childhood obesity from 1 to 6 years of age with infant feeding modalities

	No. of Participants	N (%) of obesity	ORs and 95 CIs		
			Model 1	Model 2	Model 3
***1 Years old***					
Exclusive formula feeding	5934	510 (8.6)	1.00	1.00	1.00
Mixed feeding	35,374	3077 (8.7)	1.02 (0.92, 1.23)	1.01 (0.87, 1.56)	1.07 (0.84, 1.37)
Exclusive breastfeeding	18,256	1570 (8.6)	0.99 (0.89, 1.11)	0.96 (0.83, 1.12)	0.94 (0.72, 1.21)
*P*_trend_			0.702	0.422	0.147
***2 Years old***					
Exclusive formula feeding	5934	488 (8.2)	1.00	1.00	1.00
Mixed feeding	35,374	2786 (7.9)	0.95 (0.86, 1.05)	0.92 (0.81, 1.05)	0.93 (0.74, 1.16)
Exclusive breastfeeding	18,256	1389 (7.6)	0.92 (0.82, 1.02)	**0.87 (0.75, 1.00)**	**0.78 (0.61, 0.99)**
*P*_trend_			0.108	0.045	**0.007**
***3 Years old***					
Exclusive formula feeding	5934	368 (6.2)	1.00	1.00	1.00
Mixed feeding	35,374	1666 (4.7)	**0.75 (0.66, 0.84)**	**0.70 (0.60, 0.82)**	**0.62 (0.49, 0.80)**
Exclusive breastfeeding	18,256	822 (4.5)	**0.71 (0.63, 0.81)**	**0.67 (0.57, 0.79)**	**0.57 (0.44, 0.74)**
*P*_trend_			** < 0.001**	** < 0.001**	**0.001**
***4 Years old***					
Exclusive formula feeding	5934	372 (6.3)	1.00	1.00	1.00
Mixed feeding	35,374	1759 (5.0)	**0.78 (0.69, 0.87)**	**0.78 (0.68, 0.89)**	0.79 (0.62, 1.02)
Exclusive breastfeeding	18,256	911 (5.0)	**0.78 (0.69, 0.89)**	**0.79 (0.69, 0.91)**	0.78 (0.60, 1.02)
*P*_trend_			**0.005**	**0.026**	0.188
***5 Years old***					
Exclusive formula feeding	5934	401 (6.8)	1.00	1.00	1.00
Mixed feeding	35,374	2167 (6.1)	**0.89 (0.80, 1.00)**	**0.83 (0.72, 0.97)**	0.89 (0.70, 1.13)
Exclusive breastfeeding	18,256	1074 (5.9)	**0.86 (0.76, 0.96)**	**0.76 (0.65, 0.90)**	**0.74 (0.57, 0.95)**
*P*_trend_			**0.020**	**0.002**	**0.003**
***6 Years old***					
Exclusive formula feeding	5934	612 (10.3)	1.00	1.00	1.00
Mixed feeding	35,374	3279 (9.3)	**0.88 (0.81, 0.97)**	**0.88 (0.78, 1.00)**	0.99 (0.80, 1.22)
Exclusive breastfeeding	18,256	1601 (8.8)	**0.83 (0.76, 0.92)**	**0.82 (0.71, 0.93)**	0.83 (0.67, 1.03)
*P*_trend_			**0.001**	**0.004**	**0.008**

Model 1: univariate analysis

Model 2: adjusted for sex, gestational age, birth weight and maternal characteristics (age, education, smoking status, and gestational diabetes in term pregnancy)

Model 3: adjusted for covariates in Model 2 and also for maternal prepregnancy body mass index

Testing trend for differences from exclusive formula feeding to mixed feeding to exclusive breastfeeding in each category

Compared with breastfeeding, exclusive formula feeding might be a risk factor for childhood underweight at 3 and 5 years old (Table 2). The largest association with underweight was displayed at 3 years old, with the multivariable adjusted ORs for exclusive formula feeding, mixed feeding and exclusive breastfeeding of 1.00, 0.53 (95% CI 0.33, 0.86) and 0.55 (95% CI 0.33, 0.91) (P_*trend*_ = 0.131), respectively (Model 3, Table 3).

**Table 3:** Odds ratios (ORs) of childhood underweight from 1 to 6 years of age according with infant feeding modalities

	No. of Participants	N (%) of Underweight	ORs and 95 CIs		
			Model 1	Model 2	Model 3
***1 Years old***					
xclusive formula feeding	5934	30 (0.5)	1.00	1.00	1.00
Mixed feeding	35,374	106 (0.3)	0.75 (0.48, 1.17)	0.60 (0.35, 1.02)	**0.44 (0.21, 0.91)**
Exclusive breastfeeding	18,256	73 (0.4)	0.91 (0.57, 1.46)	0.73(0.42, 1.27)	0.64 (0.31, 1.32)
*P*_trend_			0.793	0.659	0.863
***2 Years old***					
Exclusive formula feeding	5934	33 (0.6)	1.00	1.00	1.00
Mixed feeding	35,374	171 (0.5)	0.87 (0.60, 1.26)	0.77 (0.49, 1.21)	0.78 (0.40, 1.49)
Exclusive breastfeeding	18,256	98 (0.5)	0.96 (0.65, 1.42)	0.85 (0.52, 1.38)	0.86 (0.44, 1.68)
*P*_trend_			0.835	0.830	0.962
***3 Years old***					
Exclusive formula feeding	5934	70 (1.2)	1.00	1.00	1.00
Mixed feeding	35,374	290 (0.8)	**0.68 (0.52, 0.88)**	**0.59 (0.42, 0.82)**	**0.53 (0.33, 0.86)**
Exclusive breastfeeding	18,256	159 (0.9)	**0.72 (0.54, 0.95)**	0.72 (0.51, 1.03)	**0.55 (0.33, 0.91)**
*P*_trend_			0.151	0.528	0.131
***4 Years old***					
Exclusive formula feeding	5934	77 (1.3)	1.00	1.00	1.00
Mixed feeding	35,374	382 (1.1)	0.82 (0.64, 1.05)	0.84 (0.64, 1.10)	0.88 (0.51, 1.50)
Exclusive breastfeeding	18,256	197 (1.1)	0.82 (0.63, 1.07)	0.85 (0.63, 1.14)	0.95 (0.55, 1.64)
*P*_trend_			0.275	0.935	0.895
***5 Years old***					
Exclusive formula feeding	5934	94 (1.6)	1.00	1.00	1.00
Mixed feeding	35,374	376 (1.1)	**0.66 (0.53, 0.83)**	**0.62 (0.46, 0.83)**	0.65 (0.40, 1.07)
Exclusive breastfeeding	18,256	209 (1.1)	**0.72 (0.56, 0.91)**	**0.71 (0.52, 0.97)**	0.83 (0.51, 1.37)
*P*_trend_			0.090	0.302	0.735
***6 Years old***					
Exclusive formula feeding	5934	111 (1.9)	1.00	1.00	1.00
Mixed feeding	35,374	556 (1.6)	0.83 (0.67, 1.02)	0.87 (0.67, 1.14)	0.88 (0.57, 1.37)
Exclusive breastfeeding	18,256	303 (1.7)	0.88 (0.71, 1.09)	0.97 (0.73, 1.29)	1.16 (0.75, 1.81)
*P*_trend_			0.543	0.673	0.085

Model 1: univariate analysis

Model 2: adjusted for sex, gestational age, birth weight and maternal characteristics (age, education, smoking status, and gestational diabetes in term pregnancy)

Model 3: adjusted for covariates in Model 2 and also for maternal prepregnancy body mass index

Testing trend for differences from exclusive formula feeding to mixed feeding to exclusive breastfeeding in each category

Analysis of variance from marginal models for repeated measured data of categorical dependent variables were shown in Table 4. Both infant feeding modalities and ages could significantly affect childhood body size (underweight, normal weight and obesity), and there was multiplicative interaction between infant feeding modalities and ages (all *P* < 0.05).

**Table 4:** Analysis of variance from marginal models (generalized estimating equation, GEE) for repeated measured data of categorical dependent variables (obesity, normal weight and underweight)

Source	DF	Chi-Square	*P* value
Intercept	2	581,902.8	< 0.0001
Time (age)	10	1308.29	< 0.0001
Group (infant feeding modalities)	2	11.47	0.0032
Time*Group	10	136.26	< 0.0001
Residual	0		

Marginal Models were conducted to investigate changes in the dependent variable over time (1, 2, 3, 4, 5, and 6 years old), and to compare these changes across groups (breast feeding and exclusive formula feeding), and also to test whether there was multiplicative interaction between time and group

## Discussion

Based on a population-based birth cohort study in north China, the present study found that compared with exclusive formula feeding, breastfeeding was inversely associated with the risk of childhood obesity from 2 to 6 years old, and there was a trend from mixed feeding to exclusive breastfeeding. Another finding was that infant exclusive formula feeding might be a risk factor for childhood underweight at 3 and 5 years old.

The impact of breastfeeding on childhood obesity has long been under debate, with most research showing significant association [8, 9], and others showing weak or no association between breastfeeding and childhood obesity [10]. The variation in the effects of breastfeeding on childhood obesity resulted from the differences of obesity definition, breastfeeding definition, study design (cross sectional study or cohort study), residual confounding by other potential factors, and so on. In addition, the difference of children’s age across different studies might also be an influencing factor, as the association of breastfeeding with childhood obesity may be diluted over time [15]. A recent meta-analysis has shown that breastfeeding is inversely associated with a risk of early obesity in children aged 2 to 6 years (OR 0.83, 95% CI 0.73, 0.94) [16]. The present study, based on a population-based birth cohort study and with 6 times of repeated measured data, added evidence to the inverse association between breastfeeding and obesity in preschool children. In particular, the association of exclusively breastfeeding was larger as compared with that of mixed feeding. Moreover, all of the previous studies focused on the association between breastfeeding and childhood obesity, and no studies to our knowledge have assessed the association between breastfeeding and childhood underweight. The present study suggested that infant exclusive formula feeding might be a risk factor for childhood underweight at 3 and 5 years old.

Mechanistic pathways linking breastfeeding to childhood obesity are explained previously. Some researchers gave nutritional explanations. First, breast milk provides a moderate amount of calories and nutrients for infant, while formula milk provides higher levels of fat and protein than the baby’s needs [17]. Second, breast milk contains bioactive substances such as adiponectin, leptin and ghrelin, which can influence the proliferation and differentiation of the infant’s adipocytes [18], and have a protective effect against future obesity [19]. Other researchers suggested psychological and behavioral explanations. Breastfeeding can help an infant establish a healthy eating habit to control food intake, while formula feeding may teach him to neglect satiety cues [20]. Moreover, breastfed infants are more likely to delay the introduction of solid foods, which decreases the odds of childhood obesity [21].

To our knowledge, no studies have focused on the mechanism of breastfeeding on childhood underweight. Some studies suggested that lactation could promote the proliferation of beneficial bacteria including Bifidobacteria [22], and more than 6 months of exclusive breastfeeding could reduce significantly the risk for episode of gastrointestinal infection(s) during months 1–9 [23]. Based on these studies, we hypothesized that Children who are exclusively breastfed have a better gastrointestinal microenvironment and less gastrointestinal infection, and therefore are less likely to be underweight. The mechanism of breastfeeding on childhood underweight should be further investigated.

Breast milk is considered the ideal food for infants, as it provides adequate energy and nutrients to meet the infants’ needs [7]. Breastfeeding has long-term benefits throughout a child’s lifetime [7]. The WHO recommends infants should be exclusively breastfed for the first 6 months of life, and continue to breastfeed supplemented with additional foods for the first 2 years and beyond [7]. However, nearly 2 out of 3 infants are not exclusively breastfed for the recommended 6 months-a rate that has not improved in 2 decades [7]. In China, the crude and weighted exclusive breastfeeding rate under 6 months was 20.7% and 18.6% based on a national representative survey in 2013 [24]. The present study reported the rate of exclusive formula feeding, mixed feeding, and exclusive breastfeeding were 10.0%, 59.4% and 30.6%, respectively. As shown in Table 1, both maternal (age, BMI, education, smoking status, and history of gestational diabetes) and child characteristics (sex, birth weight, and gestational age) can affect the prevalence of breastfeeding. Other affecting factors include maternal race/ethnicity, breast diseases, inadequate breast milk production, employment, length of maternity leave, inadequate knowledge regarding breastfeeding, lack of familial and societal support, and lack of guidance and encouragement from health care professionals [16, 25]. To strengthen breastfeeding practices, families, employers, professional workers and society as a whole should fully support breastfeeding mothers.

The major strength is that, to our knowledge, we are the first to report the association of breastfeeding with childhood body size based on a population-based birth cohort study and with 6 times of repeated measured data. We are also the first to evaluate the association between breastfeeding and childhood underweight. Moreover, all the information used in the present study was collected from the electronic medical records, which can avoid the recall bias. Our study has certain limitations. First, almost 70% of the children screened were excluded as they lacked some of the key information. Though we compared the general characteristics between the included and excluded children, we could not exclude the possibility that the observed effect sizes in our study had departure from the true value. Second, we do not have the information of infant feeding modalities after 6 months old, so we cannot evaluate the association between the duration of breastfeeding and childhood obesity/underweight. Third, even though our analyses adjusted for an extensive set of confounding factors, residual confounding due to unmeasured factors cannot be excluded, including maternal information (chronic diseases and/or medication, employment, marital status, living alone or co-habiting, etc.) and child information (method of child birth, proportion of preterm births, need for neonatal intensive care unit, chronic diseases and/or anomalies, and childhood physical activity and dietary factors, etc.).

## Conclusion

We performed a population-based birth cohort study with 6 times of repeated measured data that targeted preschool-aged children and reported that breastfeeding was inversely associated with the risk of childhood obesity from 2 to 6 years old, and there was a trend from mixed feeding to exclusive breastfeeding. We also suggested that infant exclusive formula feeding might be a risk factor for childhood underweight at preschool time.

## Data Availability

The dataset used during the current study is available from the corresponding author on reasonable request.
